# Tasteful Brands: Products of Brands Perceived to be Warm and Competent Taste Subjectively Better

**DOI:** 10.5334/pb.bf

**Published:** 2015-06-09

**Authors:** Boyka Bratanova, Nicolas Kervyn, Olivier Klein

**Affiliations:** 1 Research Centre for Social and Intercultural Psychology, Université Libré de Bruxelles, Bruxelles, Belgium; 2 Dundee Business School, Abertay University, Dundee, UK; 3 Center Emile Bernheim, Solvay Brussels School of Economy and Management, Université Libré de Bruxelles, Bruxelles, Belgium; 4 Louvain School of Management, Catholic University of Louvain, Louvain-la-Neuve, Belgium

**Keywords:** warmth & competence, subjective taste, brand perception

## Abstract

Using survey and experimental data, the present research examines the effect of brand perception on experienced taste. The content of brand perception can be organized along the two social perception dimensions of warmth and competence. We use these two dimensions to systematically investigate the influence of brand perception on experienced taste and consumer behavior toward food products. The brand’s perceived warmth and competence independently influenced taste, both when it was measured as a belief and as an embodied experience following consumption. Taste mediated the link between brand’s warmth and competence perceptions and three consumer behavioral tendencies crucial for the marketing success of brands: buying intentions, brand loyalty, and support for the brand.

Taste is one of the most important factors influencing preference for and purchase of food and beverage products, often outweighing the role of other important determinants, such as cost and healthiness (Glanz, Basil, Maibach, Goldberg, & Snyder, 1998; Shepherd & Sparks, 1994). Rather than being solely determined by the food’s chemosensory properties, the subjective experience of taste can be affected by extraneous cues (for a review, see Coppin & Sander, 2011). Previous research has shown that product characteristics, such as the food or beverage’s price (Plassmann, O’Doherty, Shiv, & Rangel, 2008), the presence of evocative verbal labels (Wansink, van Ittersum, & Painter, 2005), and the presense of certain (non-) appetizing ingredients (Siegrist & Cousin, 2009) indeed influence consumers’ experience of the food’s taste. A possible explanation of these effects is consumers’ expectations that food with high price, described as succulent, and containing appetizing ingredients is likely to be of high quality, and therefore of superior taste. Taste expectations in turn have been shown to bias the chemosensory experience of taste when the food is consumed (Plassmann et al., 2008; Siegrist & Cousin, 2009).

The research reviewed above used manipulations of ad-hoc brand characteristics that are quite proximal to the food product, providing information about the product itself rather than the brand more broadly. Our first goal in this research is to implement a more systematic manipulation of brand perception in order to allow for more generalizable conclusions on the influence of brand perception on perceived taste and consumer behavior. To do so we use the Brands as Intentional Agents Framework (Kervyn, Fiske, & Malone, 2012). This model proposes to organize brand perception along the two social perception dimensions of warmth/intentions and competence/ability. Warmth and competence are central dimensions for organizing impressions of persons and social groups (Cuddy, Fiske, & Glick, 2008; Fiske, Cuddy, & Glick, 2007; Kervyn, Yzerbyt, & Judd, 2011). Warmth reflects perceptions of the extent to which a person or group is well-intentioned, pro-social, sincere, and trustworthy. Competence, on the other hand, reflects beliefs about a person or group’s ability and efficiency to achieve their goals. Indeed, numerous studies have demonstrated that people readily assess persons and groups based on these fundamental dimensions (Willis & Todorov, 2006), and construct their interactions accordingly (Fiske et al., 2007; Russell & Fiske, 2008). Furthermore, social perception researchers have found that similar pairs of dimensions underlie the perception of individuals (Abele, 2003; Wojciszke, 1994), countries (Phalet & Poppe, 1997; Poppe & Linssen; 1999), and cultures (Markus & Kitayama, 1991; Oyserman, Coon, & Kemmelmeier, 2002). Although these different lines of research use different names for the two dimensions and slightly different definitions, Abele and Wojciszke (2007) have shown that they all are very similar and explain upwards of 80% of the variance in social perception (Wojciszke, Bazinska, & Jaworski, 1998). There is thus ample evidence that warmth and competence are two fundamental dimensions that underlie social perception (For a review see Abele, Cuddy, Judd, & Yzerbyt, 2008).

Research has also shown that the warmth and competence dimensions also constitute a useful tool to understand the way consumers perceive different brands (Aaker, Vohs, Mogilner, 2010). Building on the notion of consumer-brand relationship (Fournier, 1998, 2009), with the Brands as Intentional Agents Framework, Kervyn et al. (2012; Fiske, Malone, & Kervyn, 2012) argued that just as when encountering a person or a group, when thinking about a brand, people try to assess how well-intentioned and transparent it is, and how able it is to achieve its goals. In the US context, these authors showed that different brands, such as Campbell’s, Porsche, and USPS, are rated differently on the warmth/intention and competence/ abilities dimensions and that these ratings are correlated with self-reported buying intentions and brand loyalty. Popular brands such as Campbell’s and Hershey’s are perceived as both well-intentioned and high on ability, subsidized brands such as Amtrak and USPS and NGOs are perceived as well-intentioned and warm but low on competence, and luxury brands such as Porsche and Rolex are high on competence but low on warmth (Aaker et al., 2010; Kervyn et al., 2012). Finally, brands such as BP and AIG that had been in the media at the time of data collection because of corporate scandals were perceived as low in both warmth and competence (Kervyn et al., 2012; Kervyn, Chan, Malone, Korpusik, & Ybarra, 2014). These survey findings were replicated experimentally. The manipulation of the intentions and ability of a fictitious brand had the predicted impact on participants’ attitude toward that brand (reported purchase intent and projected brand loyalty; Kervyn et al., 2012). Using survey and experimental data, Kervyn et al. (2014) further showed that, as for social perception, negative perceptions on the warmth/ intentions dimension are much more damaging to brand perception than negative perceptions on the competence/ability dimension. The Brands as Intentional Agents Framework thus provides a useful theoretical framework to study brand perception.

A number of other models of brand perception have already been proposed. Aaker (1997; see also Fennis, B.M. & A. Pruyn 2007; Geuens, Weijters, and De Wulf, 2009) established a brand personality measure and Grohmann (2009) added a gender approach to brand personality. The Brands as Intentional Agents Framework differs from the brand personality approach in that it has its roots in social perception literature and not in the personality model literature. We consider that when it comes to brand perception and the consumers’ buying decision, the social perception approach – trying to model how people think about others – is more pertinent than the personality model approach – trying to establish models of personality. Indeed it is the subjective way consumers think about brands (brand social perception) and not the objective way brands’ personality differ that is most determinant of the consumers’ decisions. This being said, as discussed by Digman (1990), there are commonalities between the social perception and the personality model approach, the two are therefore not mutually exclusive.

A further unexplored question is how a brand’s perceived warmth and competence influence the way consumers experience a brand’s products. This question is particularly interesting for embodied responses, such as the experience of taste when the product is a food or a beverage. It is not clear whether brand perception elements distal to the food may also alter the experience of the products’ taste. At the heart of a brand’s warmth and competence perceptions are judgments of the brand’s pro-sociality and trustworthiness, and its ability to achieve corporate and market goals. The brand’s production and distribution practices, the way it treats its customers, suppliers, and employees, or the brand’s financial performance can all be informative about the brand’s warmth and competence. Information about these aspects of the brand’s operation is substantially remote from the quality and chemosensory properties of food and beverage products, and as such present a distal source of potential influence on the experience of their taste. Nevertheless, if a brand’s warmth and competence perceptions are as central to its evaluation as they are for the evaluation of individuals and groups (Wojciszke et al., 1998), we would expect that they will influence consumers’ evaluations of all other aspects of the brand, including their experience of the taste of the brand’s products.

## Hypotheses

In the present research we test our hypothesis that distal elements of brand perception can influence experienced taste. More precisely, we predict that both elements pertaining to a brand’s perceived warmth (Hypothesis 1) and to its perceived competence (Hypothesis 2) will impact on experienced taste (Aaker et al., 2010; Coppin & Sander, 2011; Kervyn et al., 2012). We additionally test whether taste acts as a mediator of the effect of warmth and competence on behavioral tendencies important for the brand’s market success, such as buying intention (Hypothesis 3), loyalty (Hypothesis 4), and willingness to participate in an advertising campaign (Hypothesis 5). Studying buying intentions is an efficient and frequently used way to gain an understanding about actual purchase behaviour, often with a fairly good degree of accuracy; for instance, in a meta-analysis of 87 behaviours, Sheppard, Hartwick, and Warshaw (1988) found a frequency-weighted average correlation between intentions and behaviour of 0.53. Involvement with the brand and the brand’s activities, such as participation in advertising campaigns has been shown to increase consumers’ commitment to the brand and strengthen their long-term loyalty (Beatty, Kahle, & Homer, 1988). Both involvement and brand loyalty, as well as buying intentions, are therefore measures of central aspects of brand’s success; including the three measures instead of one allows for a more reliable and valid assessment of consumers’ behavioral tendencies towards the brands, especially since it allows us to go beyond self-reported opinion and obtain behavioral indication of consumers’ willingness to participate in an advertising campaign.

## Study 1

In this study we used survey data to examine whether perceptions of a water provision brand^1^ along the warmth and competence dimensions influence consumers’ evaluation of the drinking water taste. We also examined whether taste evaluations mediated the effect of warmth and competence on consumers’ brand loyalty.

The use of a non-convenience sample in this study allows us to test whether the hypothesized effects exist at the population level and ensures the ecological validity of the findings. Also, examining whether the taste of tap water varies as a function of consumers’ perceptions of the water supplying brand puts our hypothesis to a particularly stringent test. Unlike branded products, no visible cues (e.g., labels, brand logos) link tap water to the water supplying brand at the time when the water is drunk. The potential link between consumers’ perceptions of the water brand and the subjectively experienced taste of their drinking water, therefore, would reflect consumers’ long-term memory and impressions of the brand’s warmth and competence. If such evidence is found, it will present a strong case for our hypothesis that warmth and competence are fundamental dimensions for organizing impressions about brands and exert distal influence on important outcomes for the brand, as well as sensory experiences of the brand’s products, even in the absence of any proximal cues reminding of the brand’s warmth and competence at the time of consumption (e.g., like fair trade logo, or awards for quality shown on label).

### Method

The items used in the current research were administered as part of a larger survey conducted in 2009^2^. The survey examined public attitudes towards drinking water and the water supply provider in the municipality of Lilla Edet, Sweden. The whole sample consisted of 345 municipality residents. However, 40.9% (141) of these respondents did not have municipal water supplied to their home; instead, they had alternative forms of water supply, such as private wells. The remaining 59.1% (204) respondents were customers of the municipal water brand. Because the focus of the current study is evaluation of the taste of the drinking water supplied by the municipal water brand, and loyalty to that brand as a function of the brand’s perceived warmth and competence, only its customers were included in the analyses. The mean age of the subsample of 204 customers (80 female) receiving municipal water was 52.74 years (*SD* = 14.20).

It is important to note that a large part of the sample had access to alternative forms of water supply as it means that in this region the water supply provider does not have a monopoly and issues of brand perception and consumer loyalty are thus much more relevant than they are in markets where consumers only have a single option for water supply.

To measure perceived warmth (Kervyn et al., 2012), respondents were asked to rate the extent to which they agreed the water brand employees have the consumers’ interest at heart, care about the tap water quality, and have the intention to provide Lilla Edet with good water (1 = *Do not agree at all*, 9 = *Strongly agree*). Using the same scale, competence (Kervyn et al., 2012) was measured by respondents’ ratings of employees’ knowledge about the water distribution, the water plant’s equipment for purification of the water, and the technical department’s competence to maintain the water infrastructure of the municipality. Both scales were subjected to factor analysis. The three items measuring warmth loaded substantially on a single factor, which explained 74.5% of the variance. The three items measuring competence also loaded substantially on a single factor, which explained 71.1% of the variance. Both scales showed good reliability (α*_s_* ≥ .80), so the respective items were averaged to form composite measures of the water brand’s warmth and competence.

Three items were used to measure the perceived taste of the drinking water supplied by the water brand. One of them comprised of water taste rating (1 = *Not good at all*, 9 = *Excellent*). In the remaining two items respondents were asked to indicate the extent to which they agreed (1 = *Strongly disagree*; 9 = *Strongly agree*) that Lilla Edet’s water is better than the water in most other municipalities in Sweden, and that Lilla Edet’s water is better than the water in most other cities in Europe. These items were included as measures of consumers’ beliefs about the drinking water taste in light of the award for ‘tastiest water in Sweden’ granted to Lilla Edet in 2005.The three items loaded substantially on a single factor explaining 72.3% of the variance and formed a reliable scale (α = .79), so they were averaged.

To measure loyalty towards the municipal water brand, respondents were asked to rate the extent to which they would prefer to have their own well if they could, and how much they appreciated being part of the municipal water system (1 = *Not at all*; 9 = *Very much*). The two items were strongly correlated, *r*(200) = –.52, *p* < .001 and after reverse scoring preference to have own well, they were averaged in a measure of loyalty.

### Results

*Warmth and competence effects on taste.* Prior to examining the effect of brand’s perceived warmth and competence on taste, we examined the correlation between the two proposed predictors. Their correlation was high, *r*(201) = .75, *p* < .001, which posed the necessity to observe possible issues associated with multicollinearity and to test whether warmth and competence exert independent effects on the outcome variable. With these in mind, we entered warmth as a first step in a regression model, competence as a second step, and their centered interaction term as a third step. Since including the interaction term of highly correlated predictors can affect the meaning of the main effects (Judd & McCleland, 2008), we examined the effects of warmth and competence at step 2, before their interaction was introduced in the regression equation. Taste was positively predicted by both warmth, β = .32, *t*(196) = 3.75, *p* = .001, and competence, β = .34, *t*(196) = 3.99, *p* = .001, and these effects were not distorted by multicollinearity, VIF = 2.36 for warmth, and VIF = 2.36 for competence. Examining the interaction on water taste at step 3 revealed a marginally significant effect, β = –.46, *t*(196) = –1.72, *p* = .086. Unpacking the interaction through simple slope analyses further revealed that the effect of warmth on taste was stronger when the brand was perceived as low in competence (i.e., one standard deviation below the mean), B = .31, t(196) = 4.14, p < .001, and weaker when the brand was perceived as high in competence (i.e., one standard deviation above the mean), B = .17, t(196) = 1.94, p = .054.

The overall model containing the two main effects and the interaction term explained 38.7% percent of the variance associated with drinking water taste (*Adj. R^2^* = .387). To examine whether warmth and competence exerted independent effects on taste, we examined the amount of variance explained at each step of the linear regression model. Warmth alone explained 33.4%, competence explained an additional 4.6%, and their interaction 0.7%. Obtaining two significant main effects, and identifying that competence explains variance associated with taste above and beyond the variance explained by warmth demonstrate that the two dimensions do exert independent effects on water taste.

In line with the theoretical postulates of the stereotypes content model (Cuddy et al., 2008; Fiske et al., 2007; Kervyn et al., 2011) and the Brands as Intentional Agents Framework (Kervyn et al., 2012), these findings also provide empirical support that warmth and competence are substantive constructs that capture more than just valence (for additional empirical evidence, see Judd et al., 2005; Kervyn et al., 2009).

*Indirect effect of warmth and competence through taste on loyalty towards the brand.* We followed Preacher and Hayes’ (2008) approach to test the proposed indirect effect. It should be noted that contemporary approaches to mediation analysis do not require a significant effect of the IVs on the DV, and instead focus on assessing the significance of the indirect path specified by model (Hayes, 2009; Rucker, Preacher, Tormala, & Petty, 2011). To conduct a formal significance test on the specified indirect path we relied on the default bootstrapping procedure implemented in the corresponding macro for testing indirect effects (Preacher & Hayes, 2008), whereby a path is deemed significant if the 95% bias corrected bootstrap confidence intervals (based on 1000 samples) do not include zero.

**Figure 1:**
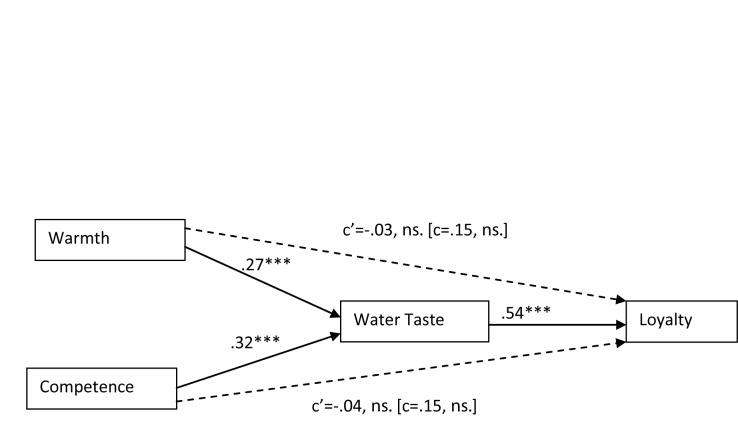
A path model depicting the indirect effects of warmth and competence on consumers’ loyalty towards the brand through taste. Path coefficients are the standardized beta coefficients. Note. *** *p*<.001.

Following Preacher and Hayes’ (2008) recommendation we conducted two separate mediation analyses testing for the indirect effect of one of the IVs through the mediator while controlling for the other IV, and vice versa. In other words, we tested for the effect of warmth (competence) on loyalty through taste while controlling for competence (warmth). The obtained effects are summarized in Figure 1. Critically for our hypothesis, water taste mediated the effects of both warmth (95% CIs between 0.05 and 0.36), and competence (95% CIs between 0.05 and 0.47) on loyalty. The overall model was significant, *F*(3,196) = 22.50, *p* < .001, explaining 26% of the variance associated with loyalty (adj. R^2^ = .26).

### Discussion

This study provides a first set of evidence of an association between a brand’s perceived warmth and competence and the evaluation of the taste of its products. Results clearly supported our first two hypotheses. Results also showed that taste mediates the link between warmth and competence perceptions and brand loyalty. Our Hypothesis 4 is thus also supported. A particular strength of this study is its ecological validity – it involved a community sample and measured naturally occurring perceptions of a brand’s warmth and competence, as well as public’s beliefs about the taste of that brand’s product. The context of tap water and water supplying brand therefore provided an especially strict test of our hypothesis. It allowed us to demonstrate that consumer’ perceptions of the brand influence how the brand’s product is experienced even in the absence of any visible or proximal cues accompanying consumption. This finding testifies for the profound influence of impressions along the fundamental dimensions of warmth and competence expert on consumers.

Nevertheless, this study also had several important limitations. First, the correlational nature of the results does not allow for strong inferences about causality. Second, the commercial context – water supply within a municipality – was unsuitable for examining consumers’ behavioural tendencies relevant for the market success of most brands, such as buying intentions. Our hypotheses 3 and 5 could therefore not be tested. Third, the measures of water taste assessed consumers’ beliefs rather than their direct sensory experience of taste and therefore failed to capture their embodied gustatory response. Study 2 was designed to address these limitations.

## Study 2

This study was designed to extend the findings from Study 1 in three important ways. First, it employed an experimental approach and manipulated perceptions of a brand’s warmth and competence to help establish the causality of their effects. Second, a description of a private food brand – chocolate producing – was used to broaden the commercial context. This allowed examining additional consumers’ behavioral tendencies, such as buying intentions (Hypothesis 3) and willingness to participate in advertising campaigns (Hypothesis 5) on top of Hypotheses 1 and 2 already tested in Study 1. Third, participants sampled the ostensible brand’s product (chocolate) and rated their taste experience. This allowed for a direct examination of the effect of the experimentally induced perceptions of the brand’s warmth and competence on consumers’ experience of its product’s taste.

### Method

*Participants.* Participants were 112 (84 female) undergraduate students from a Belgian University with a mean age of 20.57 (*SD* = 4.97). They participated in exchange for partial course credit.

*Design and procedure.* The study was based on a 2 (Warmth: warm vs. cold) × 2 (Competence: competent vs. incompetent) between-participants design. The study was introduced as examining impressions of a new brand of chocolate called ‘Morena’. Participants were informed that they would be presented with a brief description of the brand, rate their impressions of it, and then sample and evaluate the taste qualities of the chocolate produced by this brand.

To manipulate warmth (Kervyn et al., 2012), the brand was described either as a committed partner in equitable trading whose products are certified as Fair trade, or as a profit-maximizing entity, which underpays workers and purchases the cocoa from farmers who exploit child labor. To manipulate competence (Kervyn et al., 2012), the brand was portrayed as either employing highly qualified staff and consistently meeting its performance targets, or as struggling to retain its staff and failing to meet its targets (see Appendix). To check whether the descriptions influenced participants’ perceptions of the brand’s warmth and competence, they indicated the extent to which they agreed that the brand takes the public’s interest at heart, and the extent to which it is skilled and effective at achieving its goals (1 = *Strongly disagree*; 7 = *Strongly agree;* cf. Kervyn et al., 2012). Then all participants sampled the (same type of) chocolate. After sampling, participants were asked to rate how tasty (1 = *Not at all tasty*; 7 = *Very tasty*), flavorsome (1 = *Not at all flavorsome*; 7 = *Very flavorsome*), and enjoyable (1 = *Not at all enjoyable*; 7 = *Very enjoyable*) they found the chocolate. The three items loaded substantially on a single factor, explaining 80.3% of the variance, and formed a reliable scale (α = .91); therefore, they were averaged in a measure of taste. To assess buying intentions, participants were asked to indicate how likely it is that they would buy the chocolate (1 = *Not likely at all*; 7 = *Very likely*). To measure participants’ willingness to participate in the brand’s advertising campaign, which ostensibly required organizing social gatherings and giving away ‘Morena’ chocolates to friends and peers, participants were asked to leave their e-mail so that the brand can contact them. Whether participants chose to leave their e-mail was used as a behavioral measure of their willingness to support the campaign. It is important to note that the award of course credit for participation in the study did not in any way depend on the responses participants gave, including whether or not they chose to leave their email.

### Results

*Manipulation check.* The experimental manipulation of warmth and competence influenced impressions of the brand in the intended direction (Table 1).

**Table 1:** The effect of warmth and competence factors on warmth/competence manipulation check items and taste scale.

Measure	Cold M (SD)	Warm M (SD)	F(3, 108)	Cohen’s d	Incompetent M (SD)	Competent M (SD)	F(3, 108)	Cohen’s d

*Warmth:* ‘Morena’ takes the public’s interest at heart.	2.86 (1.30)	4.85 (1.20)	68.63***	1.59	3.73 (1.52)	3.91 (1.69)	0.21, ns.	0.17
*Competence:* ‘Morena’ is skilled and effective at achieving its goals.	4.19 (2.90)	4.28 (1.32)	0.01, ns.	0.04	3.27 (2.82)	5.18 (0.86)	22.96***	0.92
Taste scale	4.31 (1.14)	5.04 (1.23)	10.29**	0.62	4.41 (1.27)	4.91 (1.15)	4.63*	0.41

*Note.* Results from a 2 (Warmth: cold vs. warm) × 2 (Competence: incompetent vs. competent) ANOVA with warmth/competence manipulation check item or taste scale as the DV. The interaction terms in all analyses were non-significant (*ps* > .85). ***p < .001. **p < .01. *p < .05.

*Warmth and competence effects on taste.* Warmth and competence influenced the experience of taste independently (Table 1). As expected, participants experienced the chocolate as tastier when the brand was portrayed as warm (vs. cold) and when it was portrayed as competent (vs. incompetent). The interaction between warmth and competence was non-significant (*p* > .85).

**Figure 2:**
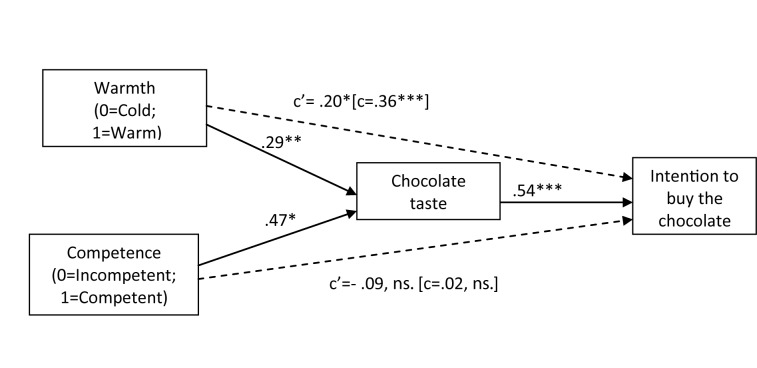
A path model depicting the indirect effects of warmth and competence on consumers’ buying intentions through taste. Path coefficients are the standardized beta coefficients. *Note:* ***p < .001. **p < .01. *p < .05.

**Figure 3:**
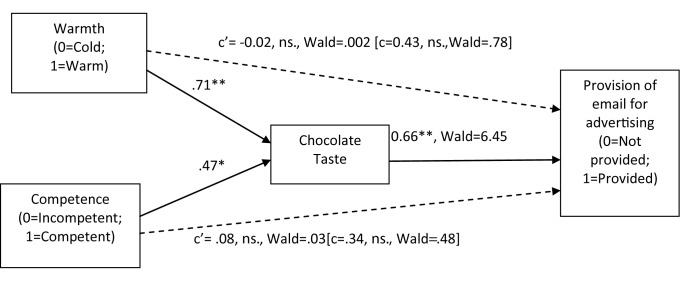
A path model depicting the indirect effects of warmth and competence through taste on participants’ willingness to participate in advertising campaign measured by provision of e-mail. Path coefficients are the unstandardized B-coefficients. *Note:* ***p < .001. **p < .01. *p < .05.

*Indirect effect of warmth and competence through taste on intentions to buy and participation in advertising.* As in Study 1, we followed Preacher and Hayes’ (2008) approach and the associated macro syntax INDIRECT to examine whether taste mediated the effect of warmth and competence on buying intentions and willingness to participate in advertising campaign as measured by participants’ provision of their e-mail. Buying intentions was measured on a Likert types scale (from 1 to 7) and it is therefore a continuous outcome variable. Whether participants provided their email was coded as a binary outcome variable (1 = email provided; 0 = email not provided). The INDIRECT macro syntax allows for the estimation of models both with continuous and binary outcome variables. Logistic regression is used for the estimation of models with binary outcome variables, and ordinary least squares regression is used for estimation of models with continuous outcome variable (for details, see http://www.afhayes.com/public/indirect.pdf). A summary of the results obtained in each analysis are presented in Figure 2 and Figure 3, respectively.

The model predicting buying intentions was significant, *F*(3, 108) = 22.66, *p* < .001, and explained 39% of the variance (adj. R^2^ = .39). Critical for our hypothesis, taste was a significant mediator of the effects of both warmth (95% CIs between 0.20 and 1.02) and competence (95% CIs between 0.02 and 0.63) on intentions to buy the chocolate. Although the magnitude of the effect of warmth on intentions to buy was reduced after the inclusion of taste, it remained significant, suggesting partial mediation.

Taste was also a significant mediator of the effect of warmth (95% CIs between 0.13 and 1.26) and competence (95% CIs between 0.04 and 0.93) on desire to participate in the brand’s advertising campaign.

### Discussion

Study 2 provides further support for our hypotheses. Both the warmth and competence dimensions exerted independent effects on taste, replicating the findings on Hypotheses 1 and 2 from Study 1. This study also extended the findings from Study 1 by showing that the effects of warmth and competence reliably emerged in a very different context (commercial chocolate brand), with a very different product, and at the level of immediate embodied response after sampling the product. Moreover, the experimental approach adopted in this study allowed us to confirm the causal role of warmth and competence perceptions in influencing taste, the consumers’ intentions to buy the product (Hypothesis 3), as well as their willingness to take part in advertising efforts (Hypothesis 5). Importantly, the influence of warmth and competence perception on these behavioral tendencies was mediated by taste perception. Furthermore, we would like to highlight that while intentions to buy the product was a self-reported measure, willingness to participate in advertising was a behavioral measure whereby participants provided their e-mail to the ostensible brand.

## General discussion

The present studies showed that consumers not only organize their perceptions of a brand along the warmth and competence dimensions (Aaker et al., 2010; Kervyn et al., 2012), but that perceptions along these dimensions have tangible consequences for consumers’ relationship with the brand and its products. While previous research has documented a positive correlation between perceiving a brand as warm and competent and reporting greater intentions to buy its products, the present research established this link causally. The main contribution of the present research is the finding that the experience of taste varies as a function of warmth and competence. To our knowledge, this is the first evidence that perceptions along these dimensions derived from information distal to the product have the capacity to alter the embodied taste experience. Evidence for this effect was obtained using both survey data and experimental design, with a non-convenience community sample and student participants, with branded product (i.e., chocolate) and natural commodity (i.e. tap water), and at the level of beliefs and as an immediate embodied experience. The widely different context in which the finding was obtained testifies for its generalizability across products and brands. Furthermore, our mediation analyses showed a previously unidentified role of taste as a mechanism linking consumers’ brand perceptions to buying intentions, brand loyalty, and behavioral support for the brand’s promotional campaign.

The present research has important implications for brand management. The finding that warmth and competence independently predict the experience of taste and consumers’ behavioural tendencies towards the brand suggests that managers of food or beverage brands can benefit in concrete terms not only from increasing the brand’s productivity and financial success, but also from engaging in and communicating about ethical and socially responsible practices. While information signaling high competence, even if it is as distal as staff retention and the brands’ financial prosperity, could potentially influence taste through creating expectations for higher food quality (cf. Plassmann et al., 2008; Siergist & Cousin, 2009), information signaling warmth is arguably unrelated to the food’s quality. An important contribution of the present research therefore is uncovering the brand’s warmth as a previously unrecognized source of positive influence on consumers’ evaluation of this brand’s products on their intentions to buy the brand’s products, and on their positive engagement with the brand. This finding is especially important in reconciling the frequently occurring misalignment between Corporate and Consumer Social Responsibility (Devinney, Auger, Eckhardt, & Birtchnell, 2006), and overcoming its detrimental effect on brands’ motivation to make bigger investments in socially responsible projects and practices. One of the obstacles to brands’ engagement in socially responsible practices is the insufficient tangible support they get from consumers for doing so (cf. (Devinney et al., 2006). Although survey data often indicate consumers’ readiness to support ethical endeavors pursuit by brands, actual purchase decisions often appear to be driven by other considerations, such as products’ quality and functional features (Devinney et al., 2006; Glanz et al., 1998). While that might be the case, our data revealed a way in which engagement in ethical and socially responsible practices can be rewarded, at least for brands producing food and beverages. Engaging in practices that portray the brand as an entity high in warmth can still have an indirect impact on consumer behavior through the impact it has on experienced taste (taste being the most important selection criteria identified for food products purchase decision, cf. Glanz et al. 1998). We stress the importance of our results on experienced taste perception. Other purely self-reported measures such as buying intentions are likely to be influenced by social desirability but experienced taste, beyond its importance for purchase decision is also less likely to be explained away by social desirability.

To summarize, our results provide a first set of evidence that aspects of brand perception distal to the product can have an impact on the experienced taste and on consumer behavior. The effects of the key dimensions – a brand’s warmth and competence – demonstrated in the two studies also showcased the utility of Brands as Intentional Agents Framework (Kervyn et al., 2012) as a theoretical model to systematically investigate phenomena related to brand perception.

### Further research

Having found that warmth and competence perception do have an impact on taste perception and consumer behavior, in further research it would be interesting to fine-tune these effects and investigate potential moderators. Does one of the two dimensions have more importance? Does that relative importance change for different outcome variables (e.g. attitudinal vs. behavioral) and/or product categories? Is the effect due to a higher than expected warmth/competence perception or to the contrary to a lower the expected perception in the low warmth/competence conditions? Does the choice context (e.g. private vs. public) have an impact? Each of these questions would be worth investigating in further research.

## Footnotes

Although brand may be unusual term to refer to a water supplier, we use ‘brand’ instead of ‘company’ to emphasize that we refer to the company’s outside image, the information that is visible to the public and creates the context which influences consumer’s experience of the product.

The survey was carried out as part of Work Area 6 of the TECHNEAU project, an integrated project funded under FP6 of the European Commission, grant number 018320.

## Appendix

### Warmth

*Warm:* ‘Morena’ is a new brand of chocolate, which is currently looking to enter the Belgian market.

The brand sources the cocoa for its products from developing countries. All products with the brand ‘Morena’ are certified as Fair Trade. The brand is a committed partner in equitable trading, ensuring that farmers in developing countries receive a better deal for their cocoa, and additional income to invest in their communities.

*Cold:* ‘Morena’ is a new brand of chocolate, which is currently looking to enter the Belgian market.

The brand sources the cocoa for its products from developing countries. In an attempt to purchase the cocoa at the lowest price possible, the brand buys from farmers who exploit child labor and pay wages that barely cover the living expenses of the workers.

### Competence

*Competent:* The brand employs highly qualified staff in all of its departments, including management, distribution and supply, as well as the some of the best experts in chocolate production. As a result, the brand consistently meets its performance and profit targets.

*Incompetent:* The brand has been experiencing difficulties in achieving its performance and profit targets. As a result, it has struggled to retain its staff; in the past few years many of its best employees have left the brand in a search for a better paid job.

## References

[1] Aaker J., Vohs K., Mogilner C. (2010). Nonprofits Are Seen as Warm and For Profits as Competent: Firm Stereotypes Matter. Journal of Consumer Research.

[2] Abele A. (2003). The dynamics of masculine-agentic and feminine-communal traits: Findings from a prospective study. Journal of Personality and Social Psychology.

[3] Abele A., Cuddy A. J. C., Judd C. M., Yzerbyt V. (2008). Fundamental dimensions of social judgment: A view from different perspectives. European Journal of Social Psychology.

[4] Abele A., Wojciszke B. (2007). Agency and communion from the perspective of self versus others. Journal of Personality and Social Psychology.

[5] Beatty S., Kahle L., Homer P. (1988). The Involvement-Commitment Model: Theory and Implications. Journal of Business Research.

[6] Coppin G., Sander D., Dolan R. J., Sharot T. (2011). The flexibility of chemosensory preferences. The neuroscience of preference and choice.

[7] Cuddy A. J. C., Fiske S. T., Glick P., Zanna M. P. (2008). Warmth and competence as universal dimensions of social perception: The Stereotype Content Model and the BIAS Map. Advances in Experimental Social Psychology.

[8] Devinney T. M., Auger P., Eckhardt G. M., Birtchnell T. (2006). The other CSR: Consumer social responsibility. Stanford Social Innovation Review.

[9] Fennis B. M., Pruyn A. (2007). You are what you wear: Brand personality influences on consumer impression formation. Journal of Business Research.

[10] Fiske S. T., Cuddy A. J. C., Glick P. (2007). Universal dimensions of social cognition: warmth and competence. Trends in Cognitive Sciences.

[11] Fiske S. T., Malone C., Kervyn N. (2012). Brands as intentional agents: Our response to commentaries. Journal of Consumer Psychology.

[12] Fournier S. (1998). Consumers and Their Brands: Developing Relationship Theory in Consumer Research. Journal of Consumer Research.

[13] Fournier S., Priester D. M. J., Park C. W. (2009). Lessons learned about consumers’ relationship with their brands. Handbook of brand relationships.

[14] Glanz K., Basil M., Maibach E., Goldberg J., Snyder D. A. N. (1998). Why Americans eat what they do: Taste, nutrition, cost, convenience, and weight control concerns as influences on food consumption. Journal of the American Dietetic Association.

[15] Hayes A. F. (2009). Beyond Baron and Kenny: Statistical mediation analysis in the new millennium. Communication Monographs.

[16] Judd C., James-Hawkins L., Yzerbyt V., Kashima Y. (2005). Fundamental dimensions of social judgment: Understanding the relations between judgments of competence and warmth. Journal of Personality and Social Psychology.

[17] Judd C. M., McClelland G. H. (2008). Data analysis: A model comparison approach.

[18] Kervyn N., Chan E., Malone C., Korpusik A., Ybarra O. (2014). Not All Disasters are Equal in the Public’s Eye: The Negativity Effect on Warmth in Brand Perception. Social Cognition.

[19] Kervyn N., Fiske S. T., Malone C. (2012). Brands as intentional agents framework: How perceived intentions and ability can map brand perception. Journal of Consumer Psychology.

[20] Kervyn N., Yzerbyt V., Judd C. (2011). When compensation guides inferences: Indirect and implicit measures of the compensation effect. European Journal of Social Psychology.

[21] Markus H., Kitayama S. (1991). Culture and the self: Implications for cognition, emotion, and motivation. Psychological Review.

[22] Oyserman D., Coon H. M., Kemmelmeier M. (2002). How American is individualism? Relational Americans and other lessons from cultural and cross-cultural research. Psychological Bulletin.

[23] Phalet K., Poppe E. (1997). Competence and morality dimensions in national and ethnic stereotypes: A study in six eastern-European countries. European Journal of Social Psychology.

[24] Plassmann H., O’Doherty J., Shiv B., Rangel A. (2008). Marketing actions can modulate neural representations of experienced pleasantness. PNAS Proceedings of the National Academy of Sciences of the United States of America.

[25] Poppe E., Linssen H. (1999). In-group favouritism and the reflection of realistic dimensions of difference between national states in Central and Eastern European nationality stereotypes. British Journal of Social Psychology.

[26] Preacher K. J., Hayes A. F. (2008). Asymptotic and resampling strategies for assessing and comparing indirect effects in multiple meadiator models. Behavior Research methods.

[27] Rucker D. D., Preacher K. J., Tormala Z. L., Petty R. E. (2011). Mediation analysis in social psychology: Current cractices and new recommendations. Social and Personality Psychology Compass.

[28] Russell A. M. T., Fiske S. T. (2008). It’s all relative: Competition and status drive interpersonal perception. European Journal of Social Psychology.

[29] Sheppard B. H., Hartwick J., Warshaw P. R. (1988). The Theory of Reasoned Action: A Meta-Analysis of Past Research with Recommendations for Modifications and Future Research. Journal of Consumer Research.

[30] Shepherd R., Sparks P., MacFie H., Thomson D. (1994). Modelling food choice. Measurement of food preferences.

[31] Siegrist M., Cousin M. E. (2009). Expectations influence sensory experience in a wine tasting. Appetite.

[32] Wansink B, van Ittersum K., Painter J. E. (2005). How descriptive food names bias sensory perceptions in restaurants. Food Quality and Preference.

[33] Wojciszke B. (1994). Multiple meanings of behavior: Construing actions in terms of competence or morality. Journal of Personality and Social Psychology.

[34] Wojciszke B., Bazinska R., Jaworski M. (1998). On the dominance of moral categories in impression formation. Personality and Social Psychology Bulletin.

